# The effect of consuming low- versus high-glycemic index meals after exercise on postprandial blood lipid response following a next-day high-fat meal

**DOI:** 10.1038/nutd.2016.26

**Published:** 2016-07-04

**Authors:** M Kaviani, P D Chilibeck, P Yee, G A Zello

**Affiliations:** 1 College of Kinesiology, University of Saskatchewan, Saskatoon, Saskatchewan, Canada; 2 College of Pharmacy and Nutrition, University of Saskatchewan, Saskatoon, Saskatchewan, Canada

## Abstract

**Background/Objectives:**

Exercise performed shortly before (that is, within half a day of) a high-fat meal is beneficial for stimulating fat oxidation after the meal and reducing postprandial triglycerides (TG). This benefit of exercise is unfortunately negated if the after-exercise food choice to replace the calories expended during exercise is one containing high-glycemic index (HGI) carbohydrates. We determined the effect of consuming low-glycemic index (LGI) carbohydrates after an exercise session on fat oxidation and TG after a subsequent high-fat meal.

**Subjects/Methods:**

Using a randomized, counterbalanced crossover design, 23 overweight or obese individuals (body mass index ⩾25 kg m^−2^) performed: walking exercise (90 min) at 1800 h followed by no meal (EX); exercise followed by a meal with LGI carbohydrates (that is, lentils, EX-LGI); exercise followed by a meal with HGI carbohydrates (that is, instant potatoes, white bread, EX-HGI); and a control condition with no exercise or meal. After a 10-h overnight fast, participants were given a standardized high-fat meal. Fat oxidation was estimated before and for 6 h after this meal from respiratory gas measures and TG determined from blood samples.

**Results:**

Fat oxidation (mean±s.d.) was higher with EX (6.9±1.7 g h^−1^) than EX-HGI (6.3±1.6 g h^−1^; *P*=0.007) and Control (5.9±1.7 g h^−1^; *P*=0.00002), and EX-LGI (6.6±1.7 g h^−1^) was higher than Control (*P*=0.002). TG total area under the curve was 18–32% lower with EX and EX-LGI compared with control (*P*=0.0005 and *P*=0.0001, respectively) and EX-HGI (*P*=0.05 and *P*=0.021, respectively).

**Conclusions:**

A meal containing HGI carbohydrates consumed after an evening exercise session cancels the beneficial effect of exercise for stimulating fat oxidation and lowering TG after a subsequent high-fat meal, whereas consuming a post-exercise meal with LGI carbohydrates retains the positive effect of exercise.

## Introduction

Postprandial lipids, the level of triglycerides (TG) after a meal, are associated with arteriosclerotic plaque formation^1^ and are a more potent predictor of cardiovascular disease risk than fasting lipids because most of an individual's day is spent in the postprandial state.^2, 3, 4^ This problem is especially evident in overweight and obese individuals who have an elevated postprandial TG response compared with lean individuals.^5^

Exercise performed within 24 h of a high-fat meal increases whole-body fat oxidation and reduces the postprandial elevation of TG in the blood;^6^ however, exercise only has this beneficial effect if it is followed by a period of fasting before the subsequent meal.^7, 8^ The effect of exercise for increasing fat oxidation and reducing postprandial TG is negated if the post-exercise food consumed to compensate for the caloric deficit elicited by the exercise is in the form of high-glycemic index (HGI) carbohydrates.^7, 8^ Performing an exercise session and then fasting could be considered an unnatural state because one usually consumes food after exercise either to enhance recovery^9^ or because exercise increases appetite.^10^ The choice of food consumed prompts varied physiological responses. HGI carbohydrates are absorbed rapidly, resulting in quick elevation of blood glucose and a large release of insulin. Conversely, the consumption of low GI (LGI) carbohydrates results in lower and more sustained levels of blood glucose and a smaller release of insulin.^11, 12^ HGI foods may attenuate the fat oxidation stimulated by exercise because insulin inhibits fat oxidation.^13^ Meals with LGI that elicit a lower insulin release have the potential to better preserve the effects of exercise for attenuating postprandial triglycerides after a subsequent meal; however, the use of LGI meals in the recovery period after exercise has never been evaluated for affecting next-day postprandial TG levels. The purpose of this study was to investigate the effect of consuming a LGI meal after an evening exercise session on next-day postprandial TG after a high-fat breakfast. It was hypothesized that compared with consuming a HGI meal post exercise, a LGI meal would elicit a lower insulin response the next day, enhance fat oxidation and attenuate the increase in TG after a high-fat breakfast.

## Materials and methods

A total of 23 participants (7 females and 16 males, 30.5±6.3 years (mean±s.d.)) with body mass index of 29.5±4.0 kg m^−2^, waist circumference of 97.6±8.7 cm and predicted maximal oxygen uptake of 35.8±4.5 ml kg^−1^ min^−1^ took part in this study. All participants were classified as ‘overweight' (that is, body mass index 25–29.9 kg m^−2^; *n*=12) or ‘obese' (body mass index ⩾30; *n*=11) but were otherwise healthy, nondiabetic, nonsmokers, with no cardiovascular conditions and were not taking any medications that could affect blood lipid or glucose levels. The study protocol was approved by the University of Saskatchewan Biomedical Research Ethics Review Board and consent was obtained from each participant before the study began. The study was registered at clinicaltrials.gov (NCT02012855).

### Experimental design

**Figure 1:**
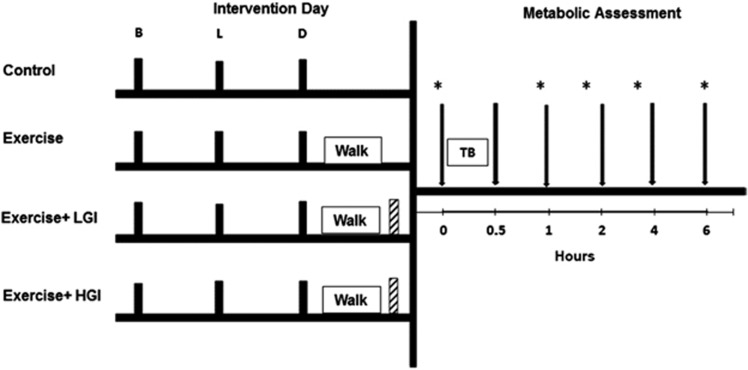
Experimental design. Participants completed four 2-day trials. Day 1 of the 2-day trial was either: a control condition; exercise with no caloric replacement; exercise followed by a low-glycemic index meal (EX-LGI); or exercise followed by a high-glycemic index meal (EX-HGI). The solid bars labeled ‘B', ‘L' and ‘D' correspond to standardized moderate-glycemic index breakfast, lunch and dinner given on day 1. The ‘Walk' corresponds to a 90-min session of walking exercise. The hatched bar following the exercise session corresponds to either a LGI or HGI meal (matched for macronutrients) containing the amount of calories expended during the exercise session. ‘TB' on day 2 corresponds to a high-fat test breakfast. The arrows (↓) correspond to blood collection for assessment of lipids, insulin and glucose and * corresponds to time points for respiratory gas collection to assess fat oxidation. Study design adapted from Burton *et al.*^7^

Following preliminary testing, each participant underwent four 2-day trials with at least a week between each 2-day trial in a randomized, counterbalanced order. Day 1 of the 2-day trials consisted of one of four conditions: Control, exercise without caloric replacement (EX), exercise followed by a LGI meal (EX-LGI) and exercise followed by a HGI meal (EX-HGI). Participants were instructed to record their sleep time after the first condition to replicate for the remaining conditions and reminded not to consume any foods at home before reporting to the lab for the next-day session. On day 2, metabolic responses (insulin, blood lipids, glucose and fat oxidation) were assessed before and for 6 h after a high-fat breakfast (Figure 1).

### Preliminary exercise and resting metabolic rate tests

At least a week before the test conditions, the Ebbeling treadmill test^14^ was used to estimate peak oxygen consumption and calculate the speed to evoke 50% of the estimated peak oxygen consumption. This was the target intensity used for the exercise interventions designed to simulate a typical walking exercise that might be prescribed to overweight/obese individuals. On a separate day, resting metabolic rate was measured after a 12-h overnight fast that was then multiplied by an activity factor to estimate daily energy expenditure.^15^ Following arrival to the lab, participants laid in a supine position for 10 min and then the expired air collection began for 20 min using indirect calorimetry and a ventilated hood (Vmax Series 29 Calorimeter, SensorMedics, Anaheim, CA, USA). Before each test, the flow volume sensor was calibrated with a 3-l syringe and the gas analyzers were calibrated with gas of known concentration, according to the manufacturer's instructions. Estimates of energy expenditure were provided by the software (Vmax/Sensor Medics Vision Software Version 4.3) accompanying the metabolic cart based on oxygen consumption and respiratory exchange ratio. The test–retest coefficients of variation for oxygen consumption (l min^−1^), carbon dioxide exhaled (l min^−1^), respiratory exchange ratio and resting energy expenditure (kcal per day) were 4.3%, 2.1%, 1.4% and 3.7%, respectively.

### Experimental trials

#### Day 1: experimental conditions

##### Control condition

Participants were instructed to abstain from any alcohol intake and exercise, aside from the prescribed exercise in the study, for 3 days before the morning meal that followed each intervention. On the day before the morning meal in each condition (that is, the day of the exercise or control intervention), dietary intake was controlled by giving participants three standardized meals (Figure 1). These meals were individualized to provide macronutrient composition representative of the average Canadian diet (30% of energy from fat, 50–55% carbohydrate and 15–20% protein), with 20% of energy provided at breakfast, 35% at lunch and 45% at dinner. Energy content was based on the participants' estimated caloric expenditure determined from the prior resting metabolic rate and added activity factor (× 1.55) for a typical sedentary individual.^16^ The meals were composed of foods that had a moderate GI (~56–69). As another level of control for diet before the interventions, participants were to photocopy their dietary intake for the 2 days before intervention day in order to replicate this intake for the 2 days before the other conditions.

On the intervention day of the control condition, participants reported to the lab at 1800 h to sit for a 90-min period, starting ~1 h after dinner; this was matched to the exercise times for the subsequent conditions.

##### Exercise condition

The intervention was identical to the control condition except for 90 min of walking at 50% of predicted peak oxygen uptake starting at 1800 h. This type and volume of exercise was chosen because it is typical of that prescribed to people with chronic conditions for improving blood lipids.^17^

##### Exercise condition including low/high GI meals

The intervention was identical to the exercise condition except that the expended energy during walking, plus 10% to account for the elevated metabolic rate typically observed during recovery,^18^ was replaced with either a LGI meal (GI=26), comprising boiled lentils, tomato sauce and Canola oil, or a HGI meal (GI=76) comprising instant mashed potato, egg whites and white bread (to match the macronutrient content of the LGI meal).^19^ The percentage of kcal from carbohydrates, protein and fat in each meal was ∼66%, 31% and 3%, respectively.^19^ Energy expenditure and substrate oxidation was measured during walking during the first meal-replacement condition in three separate 15 min intervals by indirect calorimetry by having participants breathe through a mouthpiece into a metabolic cart (Vmax Series 29 Calorimeter, SensorMedics) at 15–30, 45–60 and 75–90 min. Substrate oxidation was estimated using Brouwer Constants Equations.^20^ The energy expenditure and substrate oxidation were assumed to be similar during the second meal-replacement condition.

The individual meals were split into two boluses consumed immediately and 2 h after the exercise.

#### Day 2: metabolic assessment

After each of these conditions, participants reported to the laboratory the next morning after a 10-h overnight fast. Following 10 min lying in a supine position, fat oxidation was assessed by indirect calorimetry for 20 min. A fasting blood sample was then drawn from an antecubital vein.

Following the fasting measurements, participants consumed a high-fat test breakfast that contained 85.8 g fat, 105.6 g carbohydrate and 66 g protein, and 1452 kcal per 2 m^2^ body surface area, comprising processed cheese, sliced egg, English muffin, sausage patty and trans fat-free liquid margarine (SAUSAGE 'N EGG MCMUFFIN; MacDonald's, Saskatoon, SK, Canada). This meal composition was selected intentionally to elicit a significant rise in insulin and TG.^8^ Water intake was recorded in the first condition and replicated for the rest of the conditions. Participants rested in the lab throughout postprandial measurements. Blood samples were taken at 0.5, 1, 2, 4 and 6 h after the high-fat breakfast. Respiratory gases via indirect calorimetry and a ventilated hood for determination of fat oxidation were also collected for 20 min following 10 min lying in supine position starting at 40, 100, 220 and 340 min (Figure 1). Fat oxidation was calculated using Brouwer Constants Equations.^20^

Blood samples were immediately centrifuged for 15 min at 3500 r.p.m. (4 °C). Serum was separated and then stored at −80 °C before analysis. A microplate reader (Biotek Synergy HT Gen5 software; Biotek Instruments, Winooski, VT, USA) was used along with enzyme-linked immunoabsorbant assays to assess serum concentrations of TG, glucose, total cholesterol, low-density and very-low-density lipoprotein (LDL+vLDL), high-density lipoprotein (HDL) (BioAssay Systems, Hayward, CA, USA) and insulin (STELLUX Chemi Human Insulin, Alpco Diagnostics, Salem, NH, USA). Serum non-esterified fatty acids (NEFAs) were assessed using a protocol with an oleic acid standard solution as per the manufacturer's directions (NEFAHR(2), Wako Diagnostics Inc., Richmond, VA, USA). The intraassay coefficient of variation for all the noninsulin assays were <4% and <3.5% for the insulin enzyme-linked immunoabsorbant assay.

### Statistical analysis

All statistical analyses were performed using SPSS for Windows (SPSS Version 21; SPSS Inc., Chicago, DE, USA). The total area under the 6 h variable versus time curves (AUC), calculated using the trapezium rule, and the incremental AUC, calculated as the increment in AUC over baseline concentrations, were used as summary measures of the postprandial blood responses. A one-factor repeated measures analysis of variance was used to assess the area under the curve responses between conditions. A two-factor repeated measures analysis of variance was also used to assess responses between each condition over all measurement time points. When main effects or interactions were found, least significant difference *post hoc* tests were used to determine differences between pairs of means. Data are shown as means±s.d., except in figures, where means and s.e.m. were used for clarity. The α-level was set at 0.05.

## Results

### Day 1: experimental conditions

Walking time was identical across the conditions and between participants (all participants were able to walk 90 min during each exercise condition); however, the walking speed varied according to each participant's predicted peak oxygen consumption. The mean speed of walking was 6.0±0.9 km h^−1^. For the three walks, participants reported the exercise intensity as ‘fairly light' (EX-LGI: 10.0±0.3, EX-HGI: 9.5±0.5, and EX: 9.7±0.4) on the Borg scale of 6–20.^21^ Total energy expenditure, carbohydrate oxidation and fat oxidation during walking were 619±180 kcal, 103±41 g and 23±7 g, respectively.

### Day 2: metabolic assessment

When assessed across baseline and postprandial time points, there was a main effect of condition for respiratory exchange ratio, fat oxidation and carbohydrate oxidation, with no difference between conditions for total energy expenditure. There was also a main effect of time for fat oxidation and total energy expenditure (both increased over time; *P<*0.01). Energy expenditure across conditions was: Control (67±8 kcal h^−1^), EX-LGI (65±8 kcal h^−1^), EX-HGI (65±8 kcal h^−1^) and EX (65±9 kcal h^−1^). Respiratory exchange ratio was lower for EX-LGI (0.74±0.01), EX (0.74±0.01) and EX-HGI (0.76±0.01) compared with control (0.77±0.01) (*P<*0.01), and for EX-LGI and EX compared with EX-HGI (*P<*0.01). Fat oxidation was significantly higher in the EX condition (6.9±1.7 g h^−1^) than EX-HGI (6.3±1.6 g h^−1^) and control (5.9±1.7 g h^−1^) (*P<*0.05). Fat oxidation was also significantly higher in the EX-LGI (6.6±1.7 g h^−1^) than the control condition (*P<*0.05). Carbohydrate oxidation was significantly lower in the EX condition (1.0±4.0 g h^−1^) than EX-HGI (1.9±3.2 g h^−1^) and control (3.4±3.7 g h^−1^) (*P<*0.05). Carbohydrate oxidation in the EX-LGI (1.5±4.0 g h^−1^) was significantly lower than the control condition (*P<*0.01).

**Figure 2:**
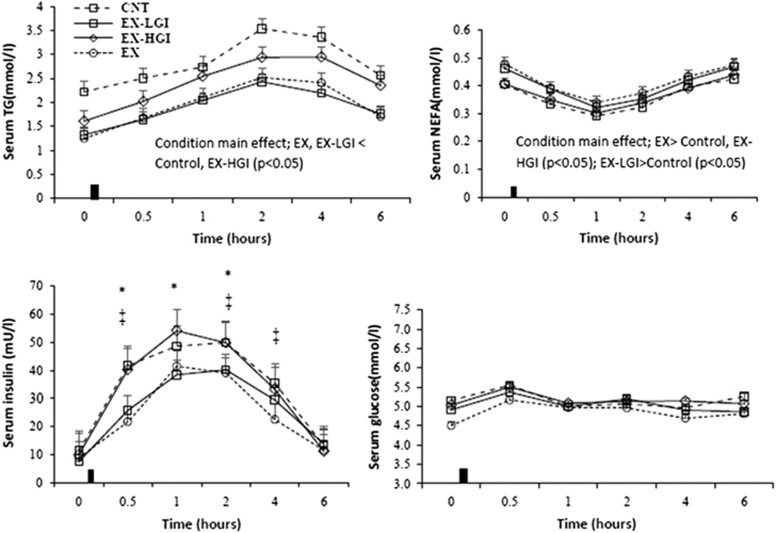
Serum TG, NEFA, insulin and glucose concentrations in the fasting state and for 6 h after consumption of the test meal in the Control, exercise+low-glycemic index meal (EX-LGI), exercise+high-glycemic index meal (EX-HGI) and exercise (EX) trials (*n*=23). The black rectangle denotes ingestion of the test meal. *EX-LGI<EX-HGI and Control (*P<*0.05), ^‡^EX<EX-HGI and Control (*P<*0.05).

**Figure 3:**
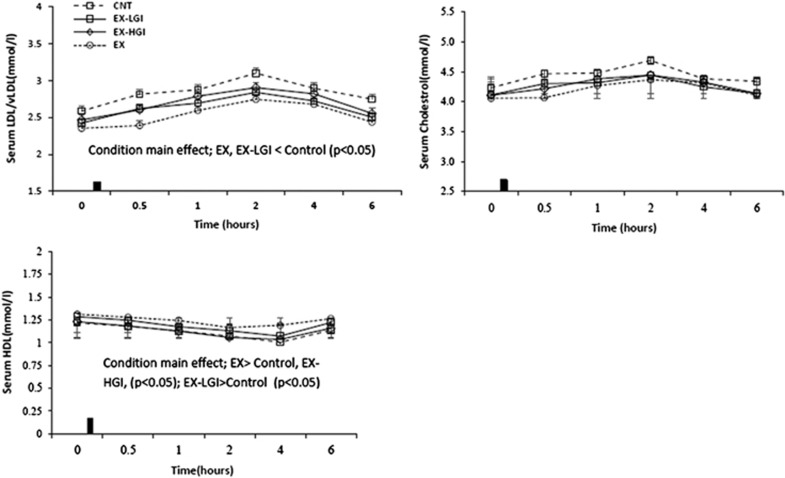
Serum LDL/vLDL, cholesterol and HDL concentrations in the fasting state and for 6 h after consumption of the test meal in the control, exercise+low-glycemic index meal (EX-LGI), exercise+high-glycemic index meal (EX-HGI) and exercise (EX) trials (*n*=23). The black rectangle denotes ingestion of the test meal.

Areas under the curve in the postprandial state are summarized in Table 1. Postprandial responses over all time points for TG, NEFA, insulin and glucose are shown in Figure 2, whereas those for other lipids (HDL, cholesterol and LDL+vLDL) are shown in Figure 3.

TG and insulin total AUC were significantly lower in EX and EX-LGI conditions versus control and EX-HGI conditions (*P*⩽0.05; Table 1). The total AUC for HDL was significantly higher in the EX condition than control and EX-HGI (*P<*0.001; Table 1). The condition main effect for total AUC for NEFA and LDL+vLDL were close to levels of statistical significance (*P*=0.059 and 0.055, respectively).

The incremental AUC did not differ significantly between conditions for any of the postprandial outcomes, with the exception of insulin that was significantly lower in EX compared with control and EX-HGI (*P<*0.01; Table 1). The condition main effect for incremental AUC for TG was close to a level of statistical significance (*P*=0.063).

When differences between conditions were assessed across baseline and postprandial time points, there were significant condition and time main effects for all outcomes except cholesterol and glucose, for which there were only time main effects (condition main effects for cholesterol and glucose had *P*-values of 0.14 and 0.059, respectively). EX-LGI and EX had lower TG compared with control and EX-HGI (*P<*0.05; Figure 2). EX had greater NEFA and HDL compared with control and EX-HGI (*P<*0.05), whereas EX-LGI was greater than control (*P<*0.05; Figures 2 and 3). EX and EX-LGI had lower LDL+vLDL compared with control (*P<*0.05; Figure 3). There was a condition × time interaction for insulin (Figure 2). EX-LGI was lower than control and EX-HGI at 0.5, 1 and 2 h after the meal (*P<*0.05). EX was lower than control at 0.5, 2 and 4 h and lower than EX-HGI at 0.5, 1, 2 and 4 h after the meal (*P<*0.05).

## Discussion

The most significant finding in this study was that a single evening 90-min exercise session of brisk walking followed by either no caloric replacement or LGI food resulted in improved baseline combined with postprandial metabolic responses to a high-fat breakfast meal the following day. Compared with HGI meal, the LGI meal after the evening exercise session elicited a lower insulin response after a high-fat breakfast the next day (Table 1 and Figure 2). Insulin inhibits fat oxidation,^13^ and therefore the lower insulin level in the EX-LGI condition could have allowed superior fat oxidation and greater clearance of TG compared with the EX-HGI condition. Although fat oxidation was not significantly different between EX-LGI and EX-HGI conditions, the EX-LGI condition was significantly higher than a control condition, whereas the EX-HGI did not differ compared with control. Lowering postprandial TG is important because this is a strong predictor for the development of cardiovascular disease.^2, 3, 4^ Our results indicate that consuming a LGI meal after an exercise session is a good strategy for lowering postprandial TG after a subsequent meal.

TG total area under the curve was significantly reduced in the EX and EX-LGI conditions compared with EX-HGI and control (Table 1). TG incremental area under the curve followed the same trend; however, the difference between conditions did not reach a level of statistical significance (*P*=0.063). This indicates that reductions in TG levels were largely mediated by reductions in baseline TG. As suggested by Burton *et al.*,^7^ this would indicate a greater effect on vLDL metabolism at the liver, with changes in meal-derived lipids making a smaller contribution to overall TG reduction. Altered hepatic vLDL metabolism to favor lower TG levels would involve direction of fatty acid flux away from re-esterification and toward oxidation.^7^ This is supported by the lower levels of LDL+vLDL in EX and EX-LGI conditions compared with control when averaged over all time points (Figure 3). Although LDL+vLDL areas under the curve were not different between conditions, total area under the curve approached a level of statistical significance (*P*=0.055), favoring lower levels in the EX and EX-LGI conditions (Table 1).

Independent of exercise, consumption of LGI foods may positively affect TG levels after a subsequent high-fat meal. Gruendel *et al.*^22^ showed that including 50 g of carob fiber derived from carob beans in a meal consumed at night by healthy individuals reduced TG level in the blood after a meal (HGI bread) consumed the next morning. Liljeberg and Björck^23^ showed that TG, glucose and insulin levels were reduced after a HGI lunch if LGI breakfast (that is, spaghetti) was fed, compared with a breakfast of HGI white bread. A meal with a LGI may reduce postprandial TG levels by several mechanisms. After a high-fat meal, TGs are initially broken down into fatty acids that are absorbed by the small intestine. They are then resynthesized into TGs and packaged mainly into chylomicrons and secreted into the circulation. A meal with a HGI stimulates the secretion of TG-containing chylomicrons into the circulation,^24^ whereas LGI carbohydrates, which have a prolonged absorption into the small intestine, do not have this same effect.^25^ Compared with carbohydrates with HGI, carbohydrates with LGI may stimulate hepatic lipase activity.^26^ An increase in hepatic lipase activity could increase lipolysis of TG bound to high-density lipoproteins and increase uptake of TG by the liver, therefore promoting a decrease in plasma TG concentration.^26^

In agreement with previous studies,^7, 8^ we showed that consuming HGI meal after an exercise session attenuates the beneficial effects of exercise for reducing postprandial TG. Exercise was also of benefit for reducing insulin levels, increasing fat oxidation and reducing LDL+vLDL and elicited higher HDL during the postprandial period compared with control, whereas EX-HGI largely negated these positive effects. Harrison *et al.*^8^ found that the exercise-induced benefit on postprandial TG was adversely affected by HGI carbohydrate replacement in the form of a glucose-containing drink. Burton *et al.*^7^ similarly found that consumption of a HGI meal replacement drink after exercise attenuated the beneficial effect of exercise for increasing fat oxidation, and reducing postprandial insulin and TG. Our results confirm these findings, but also show that consuming a LGI meal after an exercise session does not negate the beneficial effects of exercise for improving postprandial responses.

The strength of the present study was the assessment of a relatively high number of overweight/obese participants (that is, compared with other trials of exercise and postprandial lipidemia^6^). The benefit of LGI foods and exercise for reducing postprandial TGs in this population is important because overweight/obese individuals have a worse triglyceride response to a given high-fat meal compared with lean individuals.^5^ Despite our relatively high participant number, some of the analyses just missed reaching statistical significance (that is, TG incremental AUC, NEFA and LDL+vLDL total AUC, glucose averaged over time). We may have lacked statistical power to detect significant differences between conditions for these measures. Another limitation was that although we assessed LDL+vLDL, a measure of apo-lipoproteins would give a better indication of lipid particle size. A final limitation is that the nature of the study design allowed assessment of only acute responses and therefore we cannot extrapolate results over longer periods of time. Future studies will need to assess whether repeated bouts of exercise followed either by fasting or consumption of LGI foods results in longer-term alterations in blood lipid profiles. A challenge to longer-term exercise and dietary interventions is the willingness of participants to change their exercise and dietary habits.

The present study draws the attention as to how exercise if followed by LGI meal favorably affects insulin levels, fat oxidation and TG in response to a high-fat meal the following day. LGI meals consumed after exercise present more favorable metabolic responses compared with HGI meals. Consuming a LGI meal after exercise did not elicit different metabolic responses compared with the condition where no meal was consumed after exercise. We therefore propose that it is better to exercise and not eat after the exercise session. Further investigation is needed as to whether long-term feeding with LGI food following exercise could improve metabolism and possibly weight loss over the long term in overweight and obese individuals.

## Figures and Tables

**Table 1:** Postprandial responses after consumption of the high-fat breakfast

	*Control*	*EX-LGI*	*EX-HGI*	*Exercise*
*Triglyceride (mmol* *l*^−*1*^ *h)*				
Total AUC	18.4±2.6	12.5±1.5^a^^,^^b^	15.9±1.9	13.0±1.4^a^^,^^b^
Incremental AUC	5.6±1.0	4.6±0.7	6.4±0.9	5.7±0.9
				
*NEFA (mmol* *l*^−*1*^ *h)*				
Total AUC	2.2±0.1	2.4±0.2	2.3±0.1	2.5±0.2
Incremental AUC	0.14±0.046	0.10±0.04	0.16±0.08	0.10±0.05
				
*Insulin (mU* *l*^−*1*^ *h)*				
Total AUC	219.2±26.2	176.7±16.6^a^^,^^b^	215.5±21.5	159.5±11.8^a^^,^^b^
Incremental AUC	151.6±20.4	131.5±12.9	155.8±18.8	107.0±10.3^a^^,^^b^
				
*Glucose (mmol* *l*^−*1*^ *h)*				
Total AUC	30.6±4.3	30.1±3.8	30.9±3.6	29.1±3.7
Incremental AUC	1.6±0.3	2.2±0.5	1.9±0.4	2.8±0.6
				
*HDL (mmol* *l*^−*1*^ *h)*				
Total AUC	185.4±78.3	196.6±80.7	187.2±72.2	208.2±85.4^a^^,^^b^
Incremental AUC	1.1±3.2	2.5±8.5	0.5±1.3	1.7±5.6
				
*LDL+vLDL (mmol* *l*^−*1*^ *h)*				
Total AUC	580.0±36.1	538.3±34.1	552.0±36.1	521.4±35.2
Incremental AUC	81.8±14.0	80.9±14.0	77.4±17.2	74.9±11.9
				
*Cholesterol (mmol* *l*^−*1*^ *h)*				
Total AUC	765.4±34.1	734.9±34	739.3±34.9	729.6±36.9
Incremental AUC	63.2±12.7	59.6±10.7	60.3±15.9	60.1±10.4

Abbreviations: AUC, area under the 6 h concentration versus time curve; EX-HGI, exercise and high-glycemic index meal; EX-LGI, exercise and low-glycemic index meal; HDL, high-density lipoprotein; LDL, low-density lipoprotein; NEFA, non-esterified fatty acids; vLDL, very-low-density lipoprotein.

*N*=23, values are mean±s.d.

a Different versus Control (*P<*0.05).

b Different versus EX-HGI (*P*⩽0.05).
